# A Study on the Pyrolysis and Product Regulation Mechanism of Waste Polystyrene at Threshold Temperatures

**DOI:** 10.3390/molecules30030727

**Published:** 2025-02-06

**Authors:** Yong Li, Cangang Zhang, Weixuan Wang, Fengfu Yin, Wenwen Han

**Affiliations:** 1 College of Electromechanical Engineering, Qingdao University of Science and Technology, Qingdao 266061, China; ly15552797718@163.com (Y.L.); 13721910753@163.com (W.W.); 2 College of Intelligent Equipment, Dongying Vocational College of Science and Technology, Dongying 257300, China; zhangcangang@dykj.edu.cn; 3 National Engineering Research Center of Advanced Tire Equipment and Key Materials, Qingdao University of Science and Technology, Qingdao 266061, China

**Keywords:** waste polystyrene, pyrolysis characteristics, threshold temperature pyrolysis, pyrolysis products, product regulation mechanism

## Abstract

Pyrolysis technology, as a method for recycling waste polystyrenes (WPs), is widely regarded as an effective means to achieve the high value reutilization of WPs due to its environmental friendliness and the renewability of the resources used. However, in the conventional pyrolysis process for WPs, relatively high temperatures are often required to induce pyrolysis. This process not only consumes a significant amount of energy but also leads to complex and variable product compositions due to the high pyrolysis temperatures. Therefore, there is an urgent need to develop a high-value-added pyrolysis process that can lower the pyrolysis temperature of WPs and regulate its products, achieving the efficient conversion of WPs. This paper proposes a high-value “threshold temperature pyrolysis process” based on the relationships between pyrolysis temperature, threshold activation energy, and the conversion rate of WPs. The study found that under a heating rate of 10 K/min, when the conversion rate of WPs reaches 0.3, the maximum activation energy required for the entire pyrolysis process is approximately 223 kJ/mol, corresponding to a pyrolysis temperature of 673.15 K. Therefore, conducting isothermal pyrolysis at this temperature is expected to achieve the efficient conversion of WPs. The experimental results show that, compared to the conventional pyrolysis of WPs, the threshold temperature of the pyrolysis process not only lowers the pyrolysis temperature by 40 K but also regulates the distribution of pyrolysis products and the composition of pyrolysis oil, leading to a 7%wt increase in the yield of the pyrolysis oil, reaching 89.3%wt. Meanwhile, the relative content of low-molecular-weight aromatic hydrocarbons (Toluene, Styrene, and α-Methylstyrene) in the pyrolysis oil increases by 7.4%wt, which also suggests that the threshold temperature of the pyrolysis process promotes the shift in pyrolysis oil towards lighter fractions. These findings provide a solution for energy saving, emissions reductions, and the efficient conversion of WPs.

## 1. Introduction

Polystyrene (PS), with its high transparency, ease of processing, corrosion resistance, and excellent insulation properties, has widespread applications in disposable tableware, electronic product casings, packaging materials, and construction materials [1,2,3]. However, the difficulty of degrading PS and its potential for reuse have made the treatment of waste polystyrenes (WPs) an increasingly important public concern. Improper handling of WPs not only poses a serious threat to the environment and human health but also leads to resource waste [4,5,6,7]. Therefore, exploring effective methods for handling WPs to mitigate their potential negative impacts on the economy, environment, and human health is particularly crucial [8]. Currently, the recycling and reuse of WPs mainly rely on three methods: energy recovery, mechanical recycling, and chemical recycling [1]. Energy recovery aims to obtain thermal energy by incinerating WPs. However, during the incineration process, due to variations in the composition of WPs and fluctuations in the burning conditions, harmful substances such as polycyclic aromatic hydrocarbons and carbon monoxide are frequently generated [9,10,11]. On the other hand, the mechanical recycling of WPs is widely used due to its low cost and high efficiency [12,13]. However, it is important to note that after multiple cycles of mechanical recycling, the performance of PS gradually declines, and mechanical recycling is not the ultimate solution to the WP problem. In contrast, pyrolysis technology in chemical recycling can convert WPs into their original monomers or other valuable chemical feedstocks [14]. This makes pyrolysis technology one of the most promising methods for WP treatment [15,16]. It is well known that the main components of the pyrolysis products from WPs include aromatic compounds such as styrene, toluene, and ethylbenzene [17]. The relative content of these compounds is often influenced by various operating conditions, including pyrolysis temperature, holding time, heating rate, and catalyst type [18,19]. Among these factors, pyrolysis temperature plays a critical role in the pyrolysis of waste plastics, directly affecting the distribution of pyrolysis products and energy consumption. As the pyrolysis temperature increases, the energy consumption required also rises. At the same time, excessively high pyrolysis temperatures can lead to an increase in gaseous products and more active side reactions [20,21]. Therefore, lowering the pyrolysis temperature of WPs can not only effectively reduce the occurrence of side reactions, but also significantly increase the yield of pyrolysis oil. Although holding time is not the primary factor determining the distribution of WP pyrolysis products, appropriately extending the holding time can help increase the yield of styrene [22]. This provides an effective means for the efficient recovery of high-value styrene monomers. In addition, the heating rate is an important factor influencing the pyrolysis products of WPs. A slow heating rate promotes the production of pyrolysis oil and increases the content of aromatic compounds [17,23]. This may be due to the fact that the slower heating rate allows for a more complete pyrolysis reaction, favoring the formation of more pyrolysis oils and aromatic compounds. To optimize the distribution of WP pyrolysis products and enhance their selectivity, the catalytic pyrolysis of WPs can significantly increase the yield of pyrolysis oil and the styrene content in the oil [3,24]. However, the residue from WP pyrolysis, along with some pyrolysis oil, can cover the catalyst, making it difficult to separate the catalyst from the pyrolysis system. The improper handling of these catalysts may lead to the creation of new hazardous waste issues [25]. Considering the applicability and economic viability of the industrial pyrolysis of WPs, lowering the overall pyrolysis temperature of WPs, increasing the pyrolysis oil yield, and increasing the lower molecular weight components in the pyrolysis oils have become the key issues that need to be solved currently.

This paper first conducts an in-depth analysis of the pyrolysis characteristics and pyrolysis kinetics of WPs. Based on the pyrolysis characteristics and kinetic analysis results, a correlation between the activation energy, conversion rate, and pyrolysis temperature required during the pyrolysis process of WPs was established. Based on this correlation, a high-yield “threshold temperature pyrolysis process” was proposed. The “threshold temperature pyrolysis process” refers to the point during the pyrolysis of WPs when the pyrolysis conversion rate reaches a specific value, at which the activation energy (E_α_) required for the pyrolysis reaction is at its maximum. The corresponding pyrolysis temperature at this point is referred to as the threshold temperature. This study systematically tested and characterized the “threshold temperature pyrolysis process” using a thermogravimetric analyzer, a pyrolysis reactor, and gas chromatography–mass spectrometry (GC-MS). The results indicate that this process effectively lowers the pyrolysis temperature of WPs, while increasing the yield of pyrolysis oil and the relative content of low-molecular-weight aromatic hydrocarbons in the oil. In addition, we elaborated on the mechanism for controlling the pyrolysis products under this process. This study provides an effective reference for the high-value reutilization of WPs.

## 2. Results and Discussion

### 2.1. Thermogravimetric Analysis (TGA)

**Figure 1:**
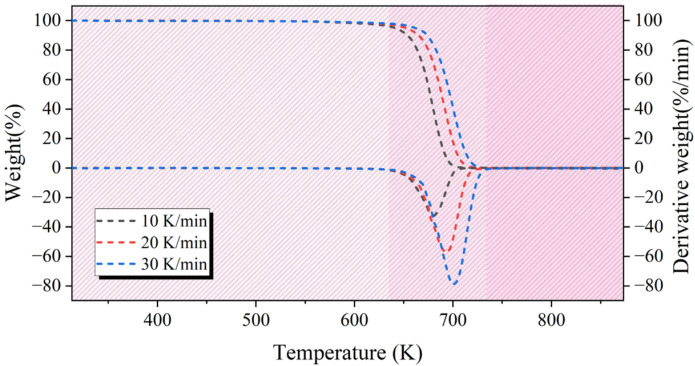
The TG and DTG curves of WPs during the pyrolysis process at three different heating rates: 10 K/min, 20 K/min, and 30 K/min.

Based on the TGA test results, the TG and DTG curves of WPs were obtained. The TG and DTG curves of WPs during the pyrolysis process at three different heating rates (10 K/min, 20 K/min, and 30 K/min) are shown in Figure 1. The pyrolysis characteristics provide further explanation of the TG and DTG curves [26]. Based on the pyrolysis characteristics of WPs, the kinetic parameters of the pyrolysis process can be calculated, and the experimental conditions for the pyrolysis reactor can be reasonably set. Table 1 lists the pyrolysis characteristics of WPs at different heating rates.

As seen in the TG and DTG curves in Figure 1, the pyrolysis of WPs is completed in a single step, with only one weight loss peak observed throughout the process. After the pyrolysis reaction ends, the remaining residue is almost zero. This indicates that the pyrolysis process of WPs is highly efficient, capable of almost completely converting WPs into pyrolysis oil and gas. Furthermore, as the heating rate increases, the TG and DTG curves of the WPs shift to higher temperature regions, and the maximum pyrolysis rate increases significantly. This phenomenon suggests that the heating rate has a significant impact on the pyrolysis characteristics of WPs and may further influence the distribution of pyrolysis products. Previous studies have confirmed that a slower heating rate contributes to the formation of a higher content of aromatic compounds during the pyrolysis of WPs [23].

Based on the data in Table 1, it can be observed that the pyrolysis termination temperature of the WPs increases as the rate of heating increases. The main reason for this phenomenon is the thermal transfer lag that occurs during the pyrolysis process [27].

### 2.2. Kinetic Analysis

**Figure 2:**
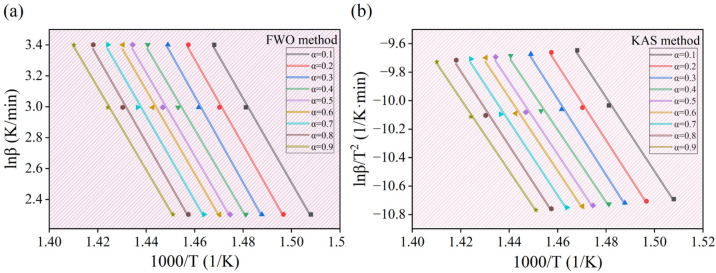
(**a**,**b**) The fitting curves for the FWO and KAS model-free methods, respectively.

The activation energy is the minimum energy required for the transformation of reactant molecules into activated molecules during the pyrolysis reaction. It is a key parameter for evaluating the pyrolysis characteristics of experimental samples. Accurately determining the activation energy *(E_α_*) at different conversion rates is crucial for understanding the pyrolysis behavior of WPs and optimizing the pyrolysis process [28,29]. The conversion rate (α) range selected for this experiment is from 0.1 to 0.9, with an interval of 0.1. The TG data of WPs at three different heating rates (10 K/min, 20 K/min, and 30 K/min) were applied to the FWO and KAS equations separately. Data fitting was then performed with 1000/T as the *x*-axis and *ln(β)*, *ln(β/T*^2^*)*, and *ln(β/T*^1.8^*)* as the *y*-axis. where β is the heating rate (K/min). Figure 2 shows the fitting curves for both the FWO and KAS model-free methods.

**Figure 3:**
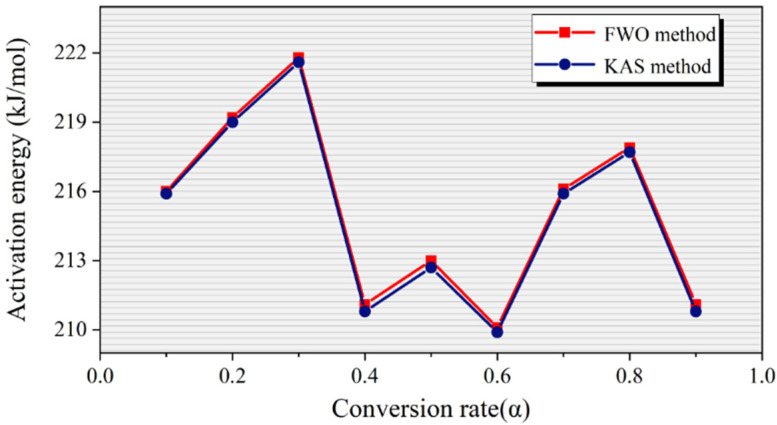
The relationship between activation energy and conversion rate calculated by two model-free methods.

Based on the slopes of the fitting curves in Figure 2, the activation energy (*E_α_*) and correlation coefficient (*R^2^*) of WPs at different conversion rates can be obtained using Equations (5) and (6). The calculation results are shown in Table 2. Additionally, Figure 3 shows the relationship between the activation energy and the conversion rate calculated by the two model-free methods.

The kinetic parameters in Table 2 indicate that the correlation coefficients (*R^2^*) calculated using both model-free methods exceed 0.999, and the activation energy (*E_α_*) values estimated by these two methods show little difference. This suggests that using model-free methods to estimate the activation energy during the pyrolysis process of WPs has a high reliability [30]. The application of model-free methods quantifies the range of the activation energy (*E_α_*) variations during the pyrolysis of WPs. Specifically, the activation energy (*E_α_*) fluctuates between 209.9 and 223.0 kJ/mol during pyrolysis. Moreover, the E_α_ values for the pyrolysis reactions at different conversion rates vary, indicating that each pyrolysis reaction at different conversion rates requires different activation energies. The difference in activation energy requirements can be attributed to the molecular structure of PS [31]. PS is formed by the polymerization of styrene monomers and contains a benzene ring in its molecular structure. The pyrolysis process of PS can be broadly classified into four stages: the initial stage, the intermediate stage, the later stage, and the final stage. In the initial stage (α = 0.1~0.3), weaker chemical bonds (such as those on the side chains) in the PS molecular chain begin to break, with a relatively low activation energy for these reactions. As the temperature rises, the depolymerization reaction of PS gradually becomes dominant, with the activation energy also increasing progressively. When the conversion rate reaches 0.3~0.6, PS transitions into the intermediate pyrolysis stage. In this stage, hydrogen transfer reactions (such as intramolecular and intermolecular hydrogen transfer) become one of the main reaction pathways, with activation energies typically lower than those of the depolymerization reaction, leading to a decrease in overall activation energy. As the conversion rate increases further to 0.8, the pyrolysis of PS enters the later stage, dominated primarily by chain termination reactions. At this stage, the pyrolysis products undergo further decomposition or secondary reactions to yield more stable products, such as carbides. These reactions require a relatively high activation energy, causing the overall activation energy to increase once again. As the pyrolysis reaction approaches completion, the remaining reactions are mainly the termination of residual free radicals. At this point, the reactant concentration is relatively low, which facilitates the reaction and leads to a decrease in activation energy.

There is a close correlation between pyrolysis temperature, activation energy, and conversion rate during the pyrolysis process of WPs. It can be observed from Figure 3 that, during the pyrolysis of WPs, when the conversion rate reaches 0.3, the activation energy (*E_α_*) required for pyrolysis reaches its maximum value of approximately 223.0 kJ/mol. The TG data show that the pyrolysis temperature corresponding to this activation energy threshold is 673.15 K. Based on the positive correlation between activation energy and temperature, it can be concluded that when the activation energy (*E_α_*) required for WP pyrolysis reaches its maximum, further pyrolysis does not require higher temperatures but can be completed by maintaining the temperature at this level. Therefore, performing isothermal pyrolysis at 673.15 K may significantly affect both the pyrolysis temperature and the final pyrolysis products.

### 2.3. Threshold Temperature Pyrolysis of WPs

**Figure 4:**
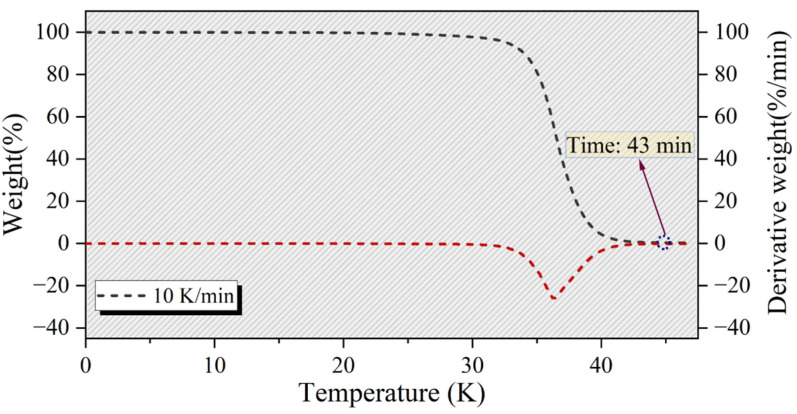
The TG and DTG curves of WPs during insulation pyrolysis at a threshold temperature of 673.15 K and a heating rate of 10 K/min.

Based on the above analysis, to determine the optimal holding time during the pyrolysis of WPs at the threshold temperature, further investigation was conducted through thermogravimetric analysis. As shown in Figure 4, the TG and DTG curves for the isothermal pyrolysis of WPs were obtained by heating at a rate of 10 K/min to the threshold temperature of 673.15 K. The experimental results indicate that when WPs were heated to 673.15 K and held for 7 min, the residual mass was not further reduced, but was maintained at 0.08 %wt, suggesting that the pyrolysis process of the WPs was complete. By comparing the TG-DTG curves of conventional pyrolysis (Figure 1) and pyrolysis at the threshold temperature (Figure 4), it can be observed that the holding temperature for threshold pyrolysis (673.15 K) is 40 K lower than the termination temperature for conventional pyrolysis (713.15 K). The decrease in pyrolysis temperature may have a significant impact on the pyrolysis products of WPs. Therefore, it is necessary to further investigate the specific effects of isothermal pyrolysis at the threshold temperature on the pyrolysis products of WPs.

### 2.4. Pyrolysis Reactor Experiment for WPs

**Figure 5:**
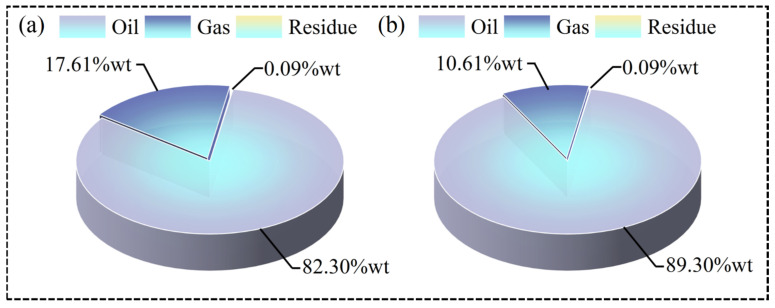
(**a**,**b**) The proportions of pyrolysis oil, pyrolysis gas, and residue at conventional and threshold temperatures, respectively.

To investigate the specific impact of the threshold temperature on the distribution of pyrolysis products, reactor experiments for both conventional pyrolysis and pyrolysis at the threshold temperature were conducted. Figure 5a,b show the proportions of pyrolysis oil, pyrolysis gas, and residue at conventional and threshold temperatures, respectively. Compared to conventional pyrolysis, the oil yield from WP pyrolysis at the threshold temperature increased by 7%wt, while the gas yield decreased by 7%wt. This indicates that pyrolysis at the threshold temperature helps to increase the yield of pyrolysis oil. In fact, lowering the pyrolysis temperature increases the yield of liquid products while decreasing the yield of gaseous products [31].

### 2.5. GC-MS Analysis of Pyrolysis Oil

**Figure 6:**
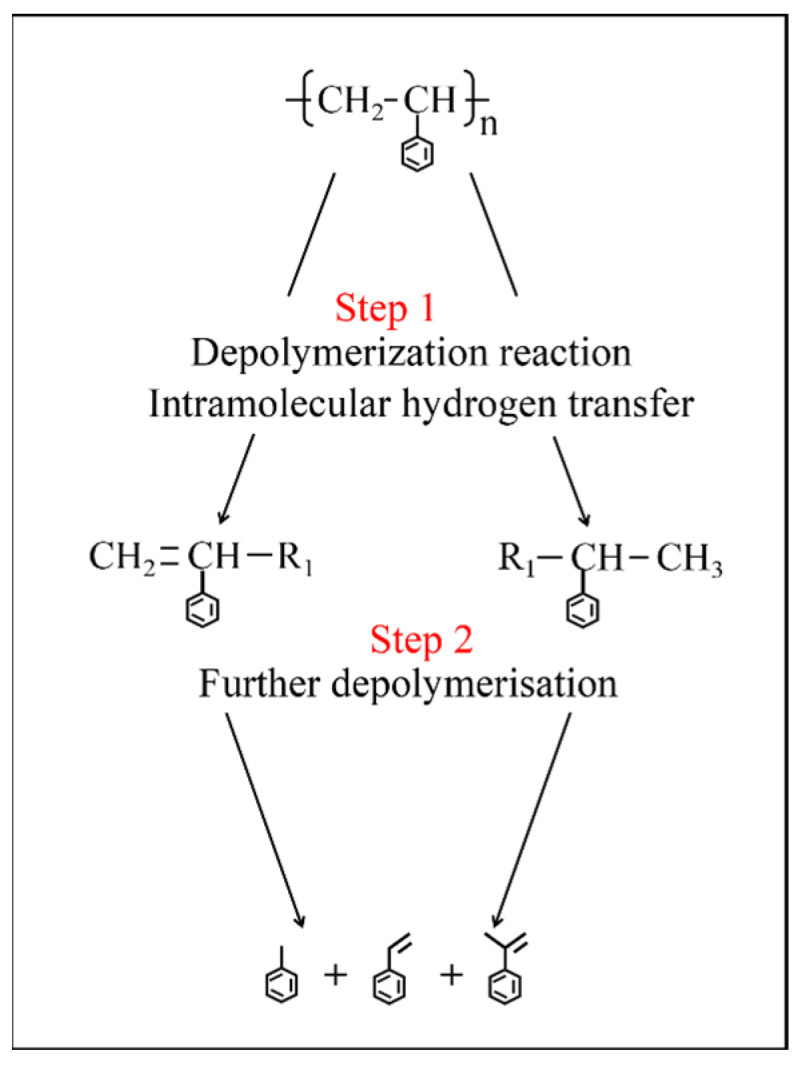
The regulation mechanism of WP pyrolysis products.

To further analyze the impact of conventional pyrolysis and pyrolysis at the threshold temperature on the main components in pyrolysis oil, this study focuses on the main components in the pyrolysis oil with a content greater than 1% and uses peak area normalization to determine their relative concentrations. Figure 6 shows the regulation mechanism of WP pyrolysis products. The analysis results show that the main products in the pyrolysis oil from both pyrolysis processes are aromatic compounds, with carbon chain lengths ranging from C_7_ to C_16_.

Table 3 lists the main components and their relative concentrations in conventional pyrolysis oil and pyrolysis oil at the threshold temperature. By comparing the two pyrolysis processes, it was found that, compared to the components in conventional WP pyrolysis oil, the contents of lower-molecular-weight compounds such as Toluene, Styrene, and α-Methylstyrene in the pyrolysis oil at the threshold temperature increased by 0.8%, 4.2%, and 2.4%, respectively, while the content of higher-molecular-weight compounds, such as Benzene, 1,1′-(3-methyl-1-propene-1,3-diyl) bis-, decreased by 9.5%. This suggests that the pyrolysis process at the threshold temperature increases the content of lower-molecular-weight aromatics, making the pyrolysis oil lighter. The increase in the content of lower-molecular-weight aromatic compounds in pyrolysis oil at the threshold temperature is primarily due to the fact that during pyrolysis, WPs first undergo depolymerization and hydrogen transfer reactions, generating PS segments with a certain molecular weight and PS segments containing C=C double bonds (Step 1). As the temperature continues to rise, when the pyrolysis temperature reaches the threshold temperature (673.15 K), the temperature no longer increases but is instead held constant at 673.15 K for isothermal pyrolysis. At this point, the holding temperature is insufficient to bring large molecular segments (such as Benzene, 1,1′-(3-methyl-1-propene-1,3-diyl) bis-) to their boiling points. Therefore, during the holding stage, these large molecular segments continue to decompose (Step 2), forming lower-molecular-weight products (such as Toluene, Styrene, and α-Methylstyrene). This indicates that performing isothermal pyrolysis at the threshold temperature can promote the conversion of more large molecular PS segments into smaller molecular segments, with the pyrolysis mechanism illustrated in Figure 6.

## 3. Materials and Methods

### 3.1. Preparation of Experimental Materials

The experimental samples used in this study were polystyrene lunch boxes, which were cleaned and dried, then pulverized using a grinder (FW177, Tester Instrument Co., Tianjin, China). The particles with a diameter smaller than 0.5 mm were selected using a standard 35-mesh sieve and were used for thermogravimetric analysis and pyrolysis reactor experiments.

### 3.2. Thermogravimetric Test

The pyrolysis behavior of the WPs was studied using a thermogravimetric analyzer (209 F3, Netzsch Company, Bavaria, Germany) over a temperature range of 313.15~873.15 K, with heating rates of 10, 20, and 30 K/min. The mass of the experimental sample was 10 mg. During the thermogravimetric testing, a purge gas flow rate of 50 mL/min (N_2_) and a protective gas flow rate of 20 mL/min (N_2_) were used. The results of the thermogravimetric analysis (TGA) provide the thermogravimetric (TG) and differential thermogravimetric (DTG) curves of the WPs, which offer information on the starting pyrolysis temperature, maximum weight loss temperature, termination temperature, residual mass, and mass change rate during the pyrolysis process [32]. In addition, the TGA data can also be used for the kinetic analysis of WPs [5], allowing the calculation of kinetic parameters (such as activation energy, correlation coefficients, etc.) at different conversion rates.

### 3.3. Calculation Formula for Kinetic Parameters

The conversion rate *(α)* during the pyrolysis of WPs is calculated using the below formula, where α represents the conversion rate (%), m_0_ is the initial mass (mg), m_t_ is the sample mass at temperature t (mg), and m_∞_ is the final residue mass (mg).

(1) $$\alpha =\frac{m_{0}-m_{t}}{m_{0}-m_{\infty }}$$

The reaction rate (dα/dt) is governed by the reaction rate constant k(T) and the pyrolysis mechanism function f(α). The relationship between k(T) and temperature T is expressed by the Arrhenius equation:

(2) $$\frac{d\alpha }{\mathrm{dt}}=k\left(T\right)f\left(\alpha \right)=\mathrm{Aexp}\left(-\frac{E_{\alpha }}{\mathrm{RT}}\right)f\left(\alpha \right)$$

where t stands for time (min), f(α) is the differential expression of the pyrolysis mechanism function, A is the pre-exponential factor (min^−1^), E_α_ denotes the activation energy (kJ/mol), and R is the universal gas constant (8.314 J/mol·K).

The relationship between T and t in a non-isothermal process is as follows:

(3) β=dT/dt

where β is the heating rate (K/min). Substituting Equation (3) into Equation (2), the comprehensive expression for the non-isothermal, non-homogeneous reaction of the test sample is obtained:

(4) $$\frac{d\alpha }{\mathrm{dT}}=\frac{A}{\beta }\exp\left(-\frac{E_{\alpha }}{\mathrm{RT}}\right)f\left(\alpha \right)$$

These model-free methods are considered an effective tool for estimating kinetic parameters. This method does not require assuming a pyrolysis mechanism function model to obtain the kinetic parameters of the experimental samples. This study employed two model-free methods to determine the kinetic parameters. This approach avoids the relative errors in the calculation process of a single model-free method, ensuring the reliability of the results [33].

The equation for the Flynn–Wall–Ozawa (FWO) method is expressed as follows [34]:

(5) $$\;\ln\left(\beta \right)=-\frac{1.052E_{\alpha }}{RT}+\ln\left(\frac{AE_{\alpha }}{RG\left(\alpha \right)}\right)$$

where *G(α)* is the integral expression of the pyrolysis mechanism function.

The equation for the Kissinger–Akahira–Sunose (KAS) method is expressed as follows [35]:

(6) $$\ln\left(\frac{\beta }{T^{2}}\right)=-\frac{E_{\alpha }}{RT}+\ln\left(\frac{AR}{E_{\alpha }G\left(\alpha \right)}\right)$$

The process of fitting curves in Equations (5) and (6) takes 1/T as the abscissa, and *ln(β)*, *ln(β/T*^2^*)*, and *ln(β/T*^1.8^*)* as the ordinate, respectively. According to the slope of the fitting curve, the activation energy (*E_α_*) and correlation coefficient (*R*^2^) at different conversion rates are obtained.

### 3.4. Pyrolysis Experiments

**Figure 7:**
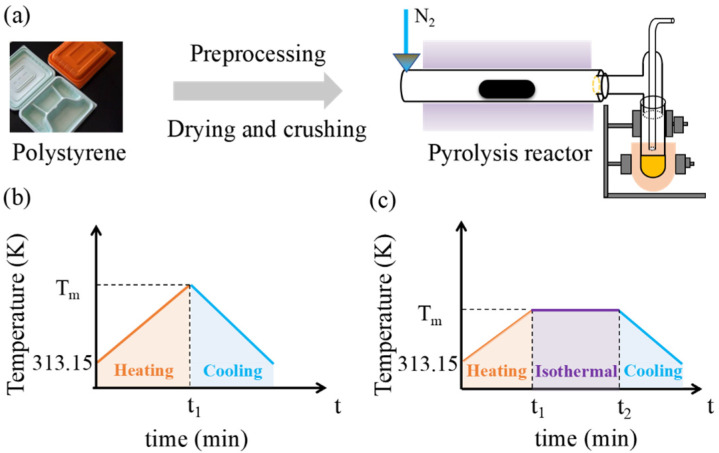
(**a**) The flowchart of the pyrolysis experiment for WPs, (**b**,**c**) the heating programs for the conventional pyrolysis experiment and the threshold temperature pyrolysis experiment for the WPs, respectively.

To investigate the regulatory effect of the threshold temperature pyrolysis process on the pyrolysis products of WPs, conventional pyrolysis experiments and threshold temperature pyrolysis experiments were conducted on WPs using a pyrolysis reactor (SK-G08123K-610, Tianjin Zhonghuan Electric Furnace Co., Ltd., Tianjin, China). The pyrolysis reactor consists of a furnace body, quartz tube, quartz boat, vacuum sealing components, and an electric control box. The carrying capacity of the quartz boat in the pyrolysis reactor is 10 g, with a sample amount of 5 g for the experimental material. The reactor has a rated power of 3 kW, a rated voltage of 220 V, and an adjustable heating rate of 0~10 K/min. The flowchart of the pyrolysis experiment for WPs is shown in Figure 7a–c which illustrate the heating programs for the conventional pyrolysis experiment and the threshold temperature pyrolysis experiment for WPs, respectively. Both pyrolysis experiments were conducted in a nitrogen environment. The flow rate of the carrier gas was 50 mL/min, and it was maintained throughout the entire pyrolysis experiment. Before each pyrolysis experiment, the initial masses of the quartz tube, quartz boat, condenser tube, and experimental sample were weighed and recorded. The heating program of the pyrolysis reactor was then set, with a heating rate of 10 K/min to the pyrolysis termination temperature (T_m_) of the WPs. After the experiment, the reactor was cooled to room temperature, and the final masses of the quartz boat, quartz tube, and condenser tube were measured. The pyrolysis oil collected in the condenser tube and quartz tube, as well as the residue in the quartz boat, were collected, and the mass and percentage of pyrolysis oil, pyrolysis gas, and residue were calculated. The mass of the pyrolysis gas was determined by subtracting the masses of the pyrolysis oil and residue from the mass of the experimental sample.

### 3.5. GC-MS Tests of Pyrolysis Oil

A gas chromatography–mass spectrometer (7890B-7000C, Agilent Technologies Company, Santa Clara, CA, USA) was utilized for the compositional analysis of the pyrolysis oil samples. The sample was first diluted with ethyl acetate (1:9) before analysis. Sampling was performed using a pipette, with a ratio of 1 part pyrolysis oil to 9 parts ethyl acetate, for a total sample volume of 1 mL, and the products were subsequently analyzed on a DB-5 column (30 m × 0.25 mm × 0.25 µm). Helium was used as the carrier gas at a flow rate of 1.0 mL/min. The oven temperature was initially set at 333.15 K and held for 2 min, then increased to 553.15 K at a heating rate of 10 K/min and held for 5 min with an injection volume of 1.0 µm. The chromatographic peaks were matched for identification using the NIST14 mass spectral library.

## 4. Conclusions

This study provides an in-depth analysis of the pyrolysis characteristics and kinetics of WPs, and establishes the relationships between pyrolysis temperature, activation energy, and conversion rate during the pyrolysis process based on these characteristics and kinetics. Based on this relationship, a high-yield “threshold temperature pyrolysis process” was proposed. Further validation of the effects of the “threshold temperature pyrolysis process” on WPs’ pyrolysis temperature and products was conducted using thermogravimetric analysis, a pyrolysis reactor, and gas chromatography–mass spectrometry (GC-MS). The results indicate that, compared to conventional pyrolysis of WPs, the threshold temperature pyrolysis process not only lowered the pyrolysis temperature of the WPs by 40 K but also increased the yield of the pyrolysis oil by 7%, reaching 89.3%. Additionally, due to the reduction in pyrolysis temperature, the relative content of lower-molecular-weight aromatic hydrocarbons (Toluene, Styrene, and α-Methylstyrene) in the pyrolysis oil increased by 7.4%. This study not only provides a new strategy for improving the efficiency of WP pyrolysis but also offers valuable insights for the resource utilization of other waste plastics. In future research, we will conduct more extensive pyrolysis experiments on WPs based on the experimental data from this study, with the aim of determining the optimal operating conditions for the industrial pyrolysis of WPs. This effort aims to promote the industrial application and dissemination of WP pyrolysis technology and to provide practical and effective solutions for the management and resource reuse of waste plastics.

## Figures and Tables

**Table 1:** The pyrolysis characteristics of WPs at different heating rates.

Heating Rate (K/min)	T_s_ (K)	T_f_ (K)	T_m_ (K)	(dα/dt)_max_ (%wt/Min)	Residue Mass (%wt)
10	660.9	677.2	713.2	25.9	0.07
20	671.3	693.3	730.7	57.4	0.07
30	679.2	700.7	738.0	78.8	0.06

Note: T_s_ is the initial pyrolysis temperature, T_m_ is the termination pyrolysis temperature, T_f_ is the peak pyrolysis temperature, and (dα/dt)_max_ is the maximum pyrolysis rate.

**Table 2:** The activation energy (*E_α_*) and correlation coefficient (*R^2^*) of WTs at different conversion rates, calculated using the FWO and KAS methods.

Conversion	FWO		KAS	
	*E_α_* (kJ/mol)	*R^2^*	*E_α_* (kJ/mol)	*R^2^*
0.1	216.0	0.9991	215.9	0.9991
0.2	219.2	0.9994	219.0	0.9993
0.3	221.8	0.9996	221.6	0.9994
0.4	211.1	0.9999	210.8	0.9999
0.5	213.0	0.9999	212.7	0.9999
0.6	210.1	0.9999	209.9	0.9998
0.7	216.1	0.9998	215.9	0.9996
0.8	217.9	0.9994	217.7	0.9994
0.9	211.1	0.9994	210.8	0.9993

**Table 3:** A list of the main components and their relative concentrations in conventional pyrolysis oil and pyrolysis oil at the threshold temperature.

Main Components of Conventional Pyrolysis Oil			
Retention Time	Component	Molecular Formula	Relative Content
2.860	Toluene	C_7_H_8_	2.5%
4.494	Styrene	C_8_H_8_	34.7%
5.908	α-Methylstyrene	C_9_H_10_	1.8%
13.697	Bibenzyl	C_14_H_14_	1.1%
15.321	Benzene, 1,1′-(1,3-propanediyl) bis-	C_15_H_16_	3.5%
16.149	benzene, 1,1′-(1-methylene-1,2-ethanediyl) bis-	C_15_H_14_	23.8%
17.172	Benzene, 1,1′-(1-butene-1,4-diyl) bis-, (Z)-	C_16_H_16_	1.1%
18.849	1-(4-Methylphenyl)-4-phenylbuta-1,3-diene	C_16_H_14_	1.1%
22.979	Benzene, 1,1′-(3-methyl-1-propene-1,3-diyl) bis-	C_16_H_16_	20.2%
**Main Components of Pyrolysis Oil at the Threshold Temperature**			
2.859	Toluene	C_7_H_8_	3.3%
4.488	Styrene	C_8_H_8_	38.9%
5.912	α-Methylstyrene	C_9_H_10_	4.2%
13.695	Bibenzyl	C_14_H_14_	1.3%
15.325	Benzene, 1,1′-(1,3-propanediyl) bis-	C_15_H_16_	3.2%
16.142	benzene, 1,1′-(1-methylene-1,2-ethanediyl) bis-	C_15_H_14_	23.2%
17.176	Benzene, 1,1′-(1-butene-1,4-diyl) bis-, (Z)-	C_16_H_16_	1.2%
18.853	1-(4-Methylphenyl)-4-phenylbuta-1,3-diene	C_16_H_14_	1.2%
22.967	Benzene, 1,1′-(3-methyl-1-propene-1,3-diyl) bis-	C_16_H_16_	10.7%

## Data Availability

Data are contained within the article.
